# Supplementary material for: Formulation of an innovative model for the bioeconomy
Source: PLoS One. 2024 Nov 4;19(11):e0309358. doi: 10.1371/journal.pone.0309358 (PMC11534262; doi:10.1371/journal.pone.0309358)
Supplement: S1 File — (PDF) [file pone.0309358.s001.pdf]

S1. Theoretical Example of DEA Application in Bioeconomy

Suppose we are evaluating the efficiency of different farms that use renewable biological resources to produce biofuels. The inputs can be land (hectares), water (liters), and fertilizers (kilograms), and the output is the amount of biofuel produced (liters).

Step 1: Theoretical Data

Step 1: Theoretical Data

Farm	Land (hectares)	Water (liters)	Fertilizers (kg)	Biofuel (liters)
A	100	2000	500	800
B	150	2500	600	1200
C	200	3000	700	1500
D	250	3500	800	1800
E	300	4000	900	2100

Step 2: Data Envelopment Analysis (DEA)

Using DEA, we calculate the technical efficiency of each farm. Let's assume we use a CRS (Constant Returns to Scale) model for simplicity.

Step 3: Theoretical DEA Results

Farm	Technical Efficiency
A	0.80
B	0.90
C	1.00
D	1.00
E	1.00

Farms C, D, and E are considered technically efficient with an efficiency score of 1. Farms A and B are not efficient, with scores of 0.80 and 0.90, respectively. This means these farms could potentially increase their biofuel production using the same amount of inputs, thereby improving their efficiency.

Illustration of DEA Application

The illustration below shows how DEA can identify which farms operate efficiently and which do not, providing a framework for improving resource allocation:

[Diagram]

```
+-------------------------------------------------+
|                  DEA Results                    |
|                                                 |
| Efficient Farms (Efficiency = 1):               |
| - Farm C                                        |
| - Farm D                                        |
| - Farm E                                        |
|                                                 |
| Inefficient Farms (Efficiency < 1):             |
| - Farm A (Efficiency = 0.80)                    |
| - Farm B (Efficiency = 0.90)                    |
|                                                 |
| Recommendations:                                |
| - Optimize resource use in farms A and B to     |
|   reach efficiency levels similar to farms C, D,|
|   and E.                                        |
+-------------------------------------------------+
```

Concluding Remark Section

DEA and SFA can be valuable tools for optimizing resource allocation in the bioeconomy, enabling better resource efficiency, profitability, and eco-efficiency. For instance, using DEA with theoretical data on farms producing biofuels, we can identify which farms are operating efficiently and which are not. The analysis revealed that farms C, D, and E are technically efficient, while farms A and B have room for improvement. By optimizing resource use in the less efficient farms, we can enhance overall resource efficiency and profitability in the bioeconomy.
