# Supplementary material for: Formulation of an innovative model for the bioeconomy
Source: PLoS One. 2024 Nov 4;19(11):e0309358. doi: 10.1371/journal.pone.0309358 (PMC11534262; doi:10.1371/journal.pone.0309358)
Supplement: S2 File — (PDF) [file pone.0309358.s002.pdf]

S2. Purpose of Mathematical Models in Bioeconomy

Mathematical models such as DEA and SFA are essential tools for analyzing and optimizing resource allocation in the bioeconomy. These models help identify inefficiencies, set benchmarks, and guide decision-making to enhance resource efficiency, profitability, and eco-efficiency.

Example Using DEA

Let's use the previous example to clarify how DEA works with theoretical data.

Theoretical Data for Farms:

Farm	Land (hectares)	Water (liters)	Fertilizers (kg)	Biofuel (liters)
A	100	2000	500	800
B	150	2500	600	1200
C	200	3000	700	1500
D	250	3500	800	1800
E	300	4000	900	2100

DEA Analysis:

Using the above data, DEA helps calculate the efficiency of each farm. The efficiency scores indicate how well each farm uses its resources to produce biofuel compared to the best-performing farms.

Results:

Farm	Technical Efficiency
A	0.80
B	0.90
C	1.00
D	1.00
E	1.00

Interpretation:

Farms C, D, and E are operating efficiently (efficiency score = 1).

Farms A and B are not fully efficient, indicating room for improvement.

Recommendations:

Farm A can increase its output or reduce its input use to improve efficiency.

Farm B should optimize its resource use to match the efficiency of Farms C, D, and E.

Example Using SFA

Stochastic Frontier Analysis (SFA) also helps in assessing the efficiency but considers random errors and statistical noise, making it suitable for complex bioeconomic environments.

Hypothetical SFA Model:

Inputs: Land, Water, Fertilizers

Output: Biofuel Production

Random Error: Measurement errors, weather conditions, etc.

Results Interpretation:

SFA results provide efficiency scores adjusted for statistical noise, offering a more realistic efficiency assessment compared to DEA.

Conclusion

The purpose of these mathematical models is to provide a structured and quantitative approach to evaluate and optimize resource use in the bioeconomy. By identifying inefficiencies and guiding improvements, DEA and SFA support sustainable and profitable bioeconomic practices.
