# Supplementary material for: Formulation of an innovative model for the bioeconomy
Source: PLoS One. 2024 Nov 4;19(11):e0309358. doi: 10.1371/journal.pone.0309358 (PMC11534262; doi:10.1371/journal.pone.0309358)
Supplement: Attachment — Submitted filename: Response to Reviewers.pdf [file pone.0309358.s004.pdf]

Wednesday 07/June/2024

Rebuttal letter

Noé Aguilar-Rivera

Editor Academico

Dear editor, thank you for all your observations, in this third round, that surely contribute to improving the quality of the research. Below I present in detail each of the improvements incorporated.

Reviewers' comments:
Reviewer #4: May 13, 2024
PLOS ONE
Reviewer Recommendation and Comments for Manuscript Number PONE-D-23-43140R1
Title: Formulation of an Innovative Model for the Bioeconomy: Unraveling the Secrets of a Sustainable Future

Reviewer Comments to Author
[1] Review summary: As proposed in the Abstract, the authors aim to "elucidate the bioeconomy's dynamic nature" and construct a comprehensive theoretical model by conducting an "extensive literature review." Their results incorporate "Georgescu-Roegen's insights." As such the Abstract is well written. However, the remainder of the manuscript does not follow the same pattern as outlined in the abstract; therefore, it is difficult to follow the entire manuscript. The subtitle (Unraveling the Secrets of a Sustainable Future) that follows the colon in Title has not really been demonstrated in the manuscript.

[1] Response to reviewer 4:
Thanks reviewer 4 for your observations, abstract was enhance marked up in green color.

Regarding to the "The subtitle (Unraveling the Secrets of a Sustainable Future) that follows the colon in Title has not really been demonstrated in the manuscript." It was eliminate as was suggesting by reviewer 6 too.

A thorough review of the manuscript was conducted for coherence based on what was outlined in the abstract. The improvements are included in green.

[2] The Introduction section attempts to define the term bioeconomy, but it fails to contextualize with their effort that specially incorporates "Georgescu-Roegen's insights" in the Introduction.

Response to the reviewer 4

Thanks for this observation, was added a paragraph in line 87-91.

Before publishing this manuscript, I recommend illustrating the Equations (1) and (2) using some theoretical data.

Dear author thanks for this observation, the ec. 17 was improvement adding the CCR-CRS and BCC-VRS model, and the adding the Figure 5, because not only is it about illustrating equations 1 and 2, but the challenge lies in integrating the 13 equations with the DEA and SFA methodology, which are the most used methods for measuring productivity and efficiency in recent decades, and integrating them into the model. Please check the figure 5, and thanks because your observation help us to improvement the equation 17.

Response to the reviewer 4

I also recommend presenting the manuscript content as they have hypothesized in the Abstract.

Response to the reviewer 4

Dear reviewer 4 thanks for this observation, it was added in the line 73-78, 83-88 y 98-99

Additional suggestions are offered below:
1. Line 95: "Ultimately, this study aims to contribute to redefining the bioeconomy to make it a more cohesive and sustainable by providing a unifying framework that takes into accommodates its diverse facets in a timeline and fosters collaboration and informed decision-making." This statement needs revision.

Response to reviewer 4:

Thanks for your observation, it was improvement: "Ultimately, this study aims to contribute to redefining the bioeconomy to make it a more cohesive and sustainable by providing a unifying framework that takes into accommodates its diverse facets over time, fostering collaboration and informed decision-making".

2. The font and font size are inconsistent in Sections 2 and 2.1.

Response to reviewer 4:
It was corrected.

3. The sentences in Line 224-226 and Line 229-230 should be revised.
Response to reviewer 4:

Dear reviewer it was improvement: Throughout the manuscript, it systematically reference the contributions of various authors who have reevaluated and reimagined the bioeconomy model proposed, thereby enriching its holistic integration [58, 61, 66, 68]. These contributions are pivotal in refining our understanding and application of the bioeconomy framework, ensuring its relevance and effectiveness in addressing contemporary challenges. Specifically, we have reconsidered the foundational principles outlined by these authors, integrating their insights to enhance the comprehensiveness and applicability of our proposed model. By acknowledging and incorporating these diverse perspectives, we strive to develop a more robust and inclusive framework for navigating the complexities of the bioeconomy landscape.

4. Line 284 citation needs to be updated.

Response to reviewer 4:
Dear reviewer 4, thanks for this observation, the cite 45 was update with 75.

5. The statement in Line 149-165 emphasizes that "The bioeconomy revolves around the use of resources that can be naturally replenished, such as crops, forests, and microorganisms ……….. It seeks to minimize the environmental impact of resource utilization, reduce greenhouse gas emissions, and promote responsible land and resource management." It would be meaningful to contrast other similar concepts and methods that utilize Data Envelopment Analysis (DEA) with what the authors have described. For example, the concept of eco-efficiency and sustainability.
Response to reviewer 4:
Thank you for your insightful observation. In response to your suggestion, we have same idea about the comparison of the bioeconomy with similar concepts such as eco-efficiency and sustainability, particularly in the context of Data Envelopment Analysis (DEA). We add this paragraph
"In summary, while the bioeconomy, eco-efficiency, and sustainability share the goal of reducing environmental impact and improving efficiency, each approaches this goal from different perspectives and with distinct focuses. DEA is a versatile tool that can be

adapted to evaluate efficiency in each of these contexts, providing a quantitative perspective on performance based on resources used and outcomes achieved".

We appreciate your valuable feedback and believe this addition enhances the clarity and depth of our manuscript.

6. The abbreviations must be spelled out at the first appearance, see Line 321 CRS.
Response to reviewer 4:
Dear reviewer it was added:" (Standard Constant Returns to Scale (CRS) and Variable Returns to Scale (VRS) models)".

7. Equation 1 and Equation 2 in Page 18 are unclear. The best way to describe these equations would be to illustrate the Q0(t) using some theoretical data.
Response to reviewer 4:

8. The literature review is incomplete. Although they have attempted to provide some definitions of bioeconomy, they fail to conduct a systematic literature review. Suggested example literature on concept of bioeconomy:
https://doi.org/10.23987/sts.69662.
Response to reviewer 4:

The cite 76 was added, thanks for this observation.

9. The statement in Concluding Remark section (Line 717-718) could be supported with the illustration of DEA using some theoretical data if realistic data is not available.
Response to reviewer 4:
Thank you for your suggestion. In response to your feedback, we have added an illustration of DEA using theoretical data to support the statement in the Concluding Remark section (Lines 717-718). This information has been included in the Supporting Information (S1) section.

10. The purpose of mathematical models should be clarified using example data.
Response to reviewer 4:
Thank you for your suggestion. To clarify the purpose of the mathematical models, we have expanded the illustration in the Supporting Information (S2) to include a detailed explanation of how Data Envelopment Analysis (DEA) and Stochastic Frontier Analysis (SFA) can be applied using example data. This expanded illustration demonstrates the practical application and benefits of these models in optimizing resource allocation and improving efficiency in the bioeconomy.

For all the above reasons, I recommend Major Revision.

Reviewer #5: The authors have incorporated most of the suggestions made by the Reviewers and therefore, the MS may be accepted for publication in PLoS ONE

Response to reviewer 5

Thanks for your observation.

Reviewer #6: This paper has carried out a deep and interesting exploration of concept and mathematical methods for "Model for the Bioeconomy", which has a good innovation. In general, the author made corresponding modifications according to the opinions of the two reviewers in the first round, and the quality of the paper was improved. But there are still some problems worth further improvement.
1) In the title, "Unraveling the Secrets of a Sustainable Future" exaggerates the significance of the content of the paper, and it is suggested that this part should be deleted or properly adjusted.

Response to Reviewer 6

Thanks for your observations, the authors following your suggest and was deleted.

2) There are too many paragraphs in many parts of the full text, so it is necessary to summarize the main arguments into a few paragraphs to enhance the readability of the article.

Response to Reviewer 6

I would like to sincerely thank you for your detailed review of our document. We have taken into account your observations and have conducted a general revision of the document to enhance its readability and clarity. Summaries of the main parts have been provided, which we hope will make the content more accessible and understandable for readers.

If you have any further suggestions or comments, please do not hesitate to let us know. We greatly appreciate your time and dedication to improving the quality of our work.

3) The aesthetics and logic of the concept figure need to be strengthened, and the relationships in Figure 1 are confusing.

Response to Reviewer 6

Thank you for your valuable feedback. We have revised and improved the aesthetics and logical clarity of Figure 1 as per your suggestion. The relationships have been reorganized to ensure they are more intuitive and easier to understand.

The updated Figure 1 now clearly illustrates the components and their relationships within the bioeconomy model, differentiating between the inputs and outputs with distinct visual elements.

4) The formula expression in the paper should be modified according to the standard, and many formulas lack necessary symbols, such as brackets.

Response to Reviewer 6

Thank you for your valuable feedback. We have carefully reviewed the expressions and formulas in the paper according to the standard conventions, ensuring the inclusion of necessary symbols such as brackets. We have made the necessary modifications to enhance clarity and precision in the mathematical expressions presented in the document.

5) The format of the full text is confused, and the title of each level is not uniform, which needs to be adjusted and standardized according to the requirements of the journal.

Response to Reviewer 6

Thank you for your review and constructive feedback on the formatting of our manuscript. We have carefully addressed your comments and made necessary adjustments to improve the clarity and uniformity of the text, particularly regarding the presentation of formulas. Here's how we've addressed your concerns:

We have thoroughly revised the presentation of formulas throughout the manuscript to ensure clarity and adherence to standard formatting conventions. Each formula is now presented in a clear and uniform manner, with all necessary symbols and notation included for better comprehension.

We have also reviewed the overall formatting of the manuscript to ensure uniformity and consistency in the title hierarchy. The titles of each section and subsection have been adjusted and standardized according to the requirements of the journal, enhancing the overall readability and organization of the text.

We believe that these improvements have significantly enhanced the presentation of the manuscript and addressed the formatting issues you raised. We appreciate your attention to detail and your commitment to maintaining the quality standards of the journal.

6) The discussion part should focus on the innovation and practical application of the new model constructed by the author.

Response to Reviewer 6

Dear Reviewer, Thank you for your insightful feedback. We have revised the discussion section to focus on the innovation and practical application of the new model constructed in the paper. The revised discussion now highlights the potential applications of DEA and SFA methodologies in optimizing resource allocation within the bioeconomy, as well as their integration into our newly constructed model.

We have emphasized the innovative aspects of our approach, particularly in addressing inefficiencies and optimizing resource allocation within the bioeconomy. Additionally, we have provided a comprehensive comparison of various bioeconomy models, including Georgescu-Roegen's foundational model, to elucidate their advantages, limitations, and applicability in diverse economic and environmental contexts.

Furthermore, we have explored how ethical and environmental considerations are integrated into the analysis of efficiency and productivity using DEA and SFA, aligning with Georgescu-Roegen's emphasis on sustainability and resource conservation.

Lastly, we have addressed the challenges in transitioning to a more sustainable bioeconomy and proposed strategies to overcome these challenges, underscoring the importance of effective public policies in fostering resilience and equity.

We believe these revisions enhance the discussion section, providing readers with valuable insights into the practical implications and innovations of our new model.

7) What is the role of "Concluding remarks"? It is suggested that many contents should be included in the introduction as an explanation of "knowledge gap" to highlight the innovation of this research.

Response to Reviewer 6

Thank you for your thorough review and valuable feedback on our manuscript. We have carefully considered each of your suggestions and made significant revisions to

improve the clarity, coherence, and focus of the paper. Here are our responses to your comments:

Introduction Enhancement: We have integrated a clearer explanation of the research's innovation and contribution to addressing the knowledge gap into the introduction. This addition highlights the significance of our study in advancing understanding and decision-making within the bioeconomy context.

Discussion Focus on Innovation and Practical Application: We have revised the discussion section to focus more explicitly on the innovation and practical application of the new model constructed in this study. By highlighting the innovative aspects and potential real-world applications of our model, we aim to provide a clearer understanding of its relevance and utility for decision-makers.

Concluding Remarks Role Clarification: We understand your suggestion regarding the role of "Concluding Remarks" and will revise this section accordingly. We will include relevant contents from the conclusion into the introduction, emphasizing the identification of the knowledge gap and the innovative aspects of our research.

Formula Expression Standardization: We have carefully reviewed the formulas in the paper and modified them according to standard formatting conventions. We have also ensured that all necessary symbols, such as brackets, are included for clarity and consistency.

Discussion Content on DEA and SFA Applications: The discussion section now provides a more detailed exploration of the potential applications of Data Envelopment Analysis (DEA) and Stochastic Frontier Analysis (SFA) in optimizing resource allocation within the bioeconomy. We have highlighted key points debatable to support this claim, including the role of DEA and SFA in uncovering inefficiencies and driving eco-efficiency.

We believe that these revisions have significantly strengthened the manuscript and addressed your concerns effectively. We appreciate your thoughtful feedback, which has undoubtedly improved the quality and impact of our research. If you have any further suggestions or require additional clarification, please don't hesitate to let us know.
