# Supplementary material for: Formulation of an innovative model for the bioeconomy
Source: PLoS One. 2024 Nov 4;19(11):e0309358. doi: 10.1371/journal.pone.0309358 (PMC11534262; doi:10.1371/journal.pone.0309358)
Supplement: Attachment — Submitted filename: Rebutal letter.pdf [file pone.0309358.s005.pdf]

Saturday 13/July/2024

Rebuttal letter

Noé Aguilar-Rivera
Academic Editor

Dear editor, thank you for all your observations, in this fourth round, that surely contribute to improving the quality of the research. Below I present in detail each of the improvements incorporated.

All the references was ordered as they appear on the each cite.

Reviewers' comments:

Reviewer's Responses to Questions

Comments to the Author
Reviewer #6: The author has made substantial replies and modifications to the questions I raised in the previous round, and the quality of the paper has been improved. After further improvement of some language and format problems, it is recommended that this paper be accepted and published.

Response: Dear reviewer 6 thanks for your contribution.

Reviewer #7: The manuscript is very interesting, it addresses a key issue in the difficulty of bioeconomic studies. The models studied are statistically strong for the topic of economics, however, for the social topic they can be subjective. The discussion could be strengthened by including literature on impact measurements in bioeconomy and policies for the growth of the bioeconomy, but in emerging countries, analyze if they exist, how they have impacted and if they favor sustainable development indices.

Spelling errors are observed in the figures of the manuscript.

Response: Reviewer #7's insightful feedback is greatly appreciated. They find the manuscript on bioeconomic studies addressing a key issue, with statistically strong models in economics but potentially subjective in social aspects. The reviewer suggests strengthening the discussion with literature on impact measurements in bioeconomy and policies for its growth in emerging countries, emphasizing their impact on sustainable development indices. Regarding the applicability to Georgescu-Roegen's bioeconomic model, while foundational in ecological economics, it primarily focuses on theoretical rather than specific policy or measurement frameworks for emerging economies. To address this, the manuscript will bolster its discussion with empirical

evidence and case studies exploring the implementation and socio-economic impacts of bioeconomic policies in diverse contexts.

The Figure 5 and 6 were improvement by the spelling errors, we give the thanks to the reviewer for this.

Bst Rgs

Carlos
