# Supplementary material for: Formulation of an innovative model for the bioeconomy
Source: PLoS One. 2024 Nov 4;19(11):e0309358. doi: 10.1371/journal.pone.0309358 (PMC11534262; doi:10.1371/journal.pone.0309358)
Supplement: Attachment — Submitted filename: Response to Reviewers.docx [file pone.0309358.s003.docx]

Wednesday 06/march/2024

Noé Aguilar-Rivera

Editor Academico

Dear editor, thank you for all your observations that surely contribute to improving the quality of the research. Below I present in detail each of the improvements incorporated.

Reviewer # 1

[1] Reviewer #1: I appreciate the possibility of reading this paper. It focuses on a topic increasingly relevant in academia and public policy.

I strongly value the mathematical development presented by the authors. The paper displays mastery of the theoretical application of DEA and SFA and how they can be used to evaluate options and make environmentally sustainable decisions.

Nonetheless, the paper is written in an unusual way for academic writing, making its readiness difficult. For instance, the introduction states the paper's goal but doesn't establish a context where this decision-making modeling can work. Later in the paper, one possible deduction is that the authors are thinking in public policy, but this is left to the readers to speculate.

Response:

Thank you for your insightful feedback on our manuscript. I have carefully reviewed your comments and made significant revisions to enhance the clarity and readiness of the paper.

Specifically, we have reworked the introduction to establish a clearer context for the proposed decision-making modeling within the realm of public policy. I have cited concrete examples and scenarios where the model could be effectively applied, particularly in the context of policymaking processes related to the bioeconomy. Additionally, I have explicitly elucidated the connection between the theoretical model and its practical implications for public policy, ensuring that readers can readily understand the relevance and significance of our work within this domain.

I believe that these revisions have substantially improved the readability and accessibility of the paper, addressing your concerns regarding the clarity of the introduction and the establishment of a context for the decision-making modeling. We appreciate your valuable feedback, which has undoubtedly strengthened the quality of our manuscript.

[2] Also, in the introduction, some bullet ideas mention critical elements from the literature review, but these elements are not further developed in the paper. Later, there is a list of the paper's contributions; however, they are not linked with other sections of the introduction or literature about the topic.

Response:

Thank you for this observation. The bullets ideas indicated in the introduction moved to the literature review section. I consider that their observation in the introduction should be focused on the contribution of this study, highlighting that the model could support decision making. to decision makers and the public agenda.

[3] Literature review: This section requires a profound review. Some constitutive elements of the concept are presented, but this is not a literature review. Here, as a reader, I expect to get information about the recent literature on bioeconomy, its applications, theoretical models, measurements, and so forth. In other words, I would like to read a review that allows me to understand the state of knowledge in this field and how this paper fits into the current literature. Additionally, due to the declaration of importance of this theory, a comprehensive explanation of Georgescu-Roegen's framework is deeply missed.

Response:

Dear reviewer 1 thanks for your observation. Regarding your observation “recent literature on bioeconomy, its applications, theoretical models” it is added in the “**Economic Theories Relevant to the Bioeconomy”, “The measurement scopes of DEA-SFA for Georgescu-Roegen's bioeconomic model**”, “**Assumptions of the DEA Model - Georgescu-Roegen's Bioeconomic Model”,** regarding to a comprehensive explanation of Georgescu-Roegen's framework is deeply missed. It is added in **Results and Discussion section**. “Georgescu-Roegen's bioeconomic model is characterized by several key principles, as outlined in the abstracts”. See it, after the Figure 3.

[4] Variables: This section presents methodological elements that must be considered when conducting such an endeavor, but it does not explain the chosen variables and the theoretical intuitions behind them.

Response:

Dear reviewer 1, thanks for this observation. Were added four paragraph for clarify it.

[5] The following sections are challenging to follow up because it is not clear its intention to develop the paper's central argument. For example, the section "Economic theories relevant to the bioeconomy" is based on one author, and it presents very well-known ideas regarding the evolution of mathematical tools in economics. It is not clear why this information would be important in this context.

Response:

Thank you for this observation. Overall, the entire document has been reviewed to ensure coherence based on the proposed research objective. Well, the author, Jiménez (2014), provides an overview of the issue surrounding economic models. The third phase, from 1960 to the present day, highlights the development of dynamic analysis and the use of stochastic processes, employed by the new classical economics and the new Keynesian economics. So it is a introduction to the DEA and SFA incorporate to the model Georgescu Rogen. In this section was added a paragraph.

[6] Finally, I strongly suggest a Discussion section. It is necessary to explain what these findings mean and how they contribute to the most recent literature. It is unclear if this model was thought for companies, countries, regions, or other subjects. Furthermore, an exploration of the model's limitations is required.

Dear reviewer 1, thanks for this observation, it is considering as Results and Discussion, where I include the most recent literature. Was added a paragraph for giving the context that you refer. Was added in the New Bioeconomy Model.

Regarding to the unclear model was thought……..it is considered in the **concluding remarks**. A paragraph was added.

Reviewer # 2

[7] Reviewer #2: This study aims to elucidate the bioeconomy's dynamic nature, constructing a comprehensive theoretical model.

It is quite interesting, with well supported data. However, there are quite many questions about the study.

Response:

Dear reviewer 2, thanks for your observation. In the conclusions section, we highlight the characteristics and gaps that any model may present. We consider that the contribution of this study encourages the challenge of identifying ways and techniques to refine the model in the socio-economic conditions of production units.

Reviewer # 3

[8] Reviewer #3: This is a confusing paper describing production functions and approaches to estimate productivity, claiming that would be a model of the Bioeconomy. It took me to read until the very end of the paper when the authors finally describe why they have presented all these equations. Most sections are completely unrelated to each other. Page numbers are missing (neither is there line numbering), making it really difficult to review. No numbering of sections, many headers the same size and font. Unclear how the paper is structured. Zero innovative content, not a scientific paper.

Response:

Dear reviewer 3 thanks for your observations. We are revised and order all the section to give the justification of each. We have put the numbers and the line numbering. Also we have put numbering to each section. Our contribution is the construction a Bioeconomy model as you can see in the literature reviewed there is a gap that we consider to fill.

[9] More detailed comments:

Introduction

- Strange spacing, paragraphs after single sentences

Response:

It was corrected.

- Strange structure, paragraphs starting with bullet points

Response:

It was corrected.

- Typo: “the review literature“

- The following paragraph is a complete repetition of the previous sentences and redundant: “In conclusion, the bioeconomy does not have a single model, but rather encompasses diverse approaches and perspectives. It is important to consider the evolution of the concept, the crosssectional nature of the sector, and the shift towards a broader perspective for a comprehensive understanding of the bioeconomy [7, 8 9].”

Response:

It was corrected. It was eliminated.

- More one to one repetitions: “The bioeconomy has been framed differently across various fields and sectors”; followed by more repetitions of the exact same sentences

Response:

It was corrected.

- The introduction is a repetition of the same arguments and the same references over and over again, using almost the exact same words, completely blown up without much content.

Response:

It was corrected.

[10] Literature review

- Figures 1 and 2 are mixed up. The first part of the lit review seems to talk about figure 2 although there is no 1-to-1 overlap of text and figure. Both figures have no real sources. Figure 1 states the source: Scopus AI data. Unclear what that is.

- Shortest literature review ever

Response:

It was corrected. Source: Scopus AI data was eliminate. Both Figures refers the key word of the Bioeconomy and the second figure refer a map of this concept.

[11] Variables

- Under the header “variables” comes a definition of different parts of models. There are no sources provided and it looks like it comes directly out of ChatGPT, because it is written by directly addressing the reader and giving a cooking recipe.

Response:

Thank you for this observation, the researchers' contributions on this topic have been better written, if the corresponding citations were added.

[12] - Economic Theories Relevant to the Bioeconomy: Suddenly change of citation style and strange paragraph on the history of economic models based on only one source

Response:

Thank you for noticing this information. Of course, in this section we wanted to start with this author's comment on how mathematically economic processes have been interpreted, realizing that in our time we are working with analysis of enveloping data. This allows us to justify in a certain way our Bioeconomy model based on stochastic processes to measure efficiency and productivity.

[13] - Assumptions of the DEA Model - Georgescu-Roegen's Bioeconomic Model: unclear listing of assumptions of DEA (Data Envelopment Analysis) and SFA (Stochastic Frontier Analysis) without the provision of sources

Response:

Dear reviewer, please check that is referenced with {37,45, 64], and remember that our model follow the consideration by author indicated in the review literature, specific Variable section.

[18]- The measurement scopes of DEA-SFA for Georgescu-Roegen's bioeconomic model: now comes a definition of DEA (Data Envelopment Analysis) and SFA (Stochastic Frontier Analysis); apparently there should some productivity measurement, but I only try to read between the lines

Response:

Dear reviewer, thanks for this observation. Of course this is correct. It was developed in the results section.

[14]- Results and discussion: strange title, completely unrelated to text, talks about Georgescu-Roegen's model of Bioeconomy, but means “bioeconomics”; some other models and concepts are mentioned without explaining them.

Response:

Dear reviewer, thanks for this observation, it was corrected. And was subdivided in Results Other section Discussion.

[20]- Again figures 3 and 4 with source: Scopus AI data. Unclear what that is. Figures not really explained, but also seem trivial

Response:

Dear reviewer, thanks for this observation, it was corrected

[15]- A function is shown for Georgescu-Roegen’s Bioeconomic Model but not really explained, sources are in Spanish

Response:

Dear reviewer, thank you for your valuable comments, in this section it is rather to briefly present Georgescu Rogen's model, because in the review of the literature it has been explained, but rather to focus on how, from the model, we extend the proposed model as a result of our research.

Dear reviewer the [31] references was added.

[16]- Then comes a description of The Constant Returns to Scale Model (CRS), DEA, some linear programming but it is all completely unclear how that relates to a comprehensive model for the bioeconomy

- 1.5) Panel Data, DEA and the Malmquist Index: more ways to measure productivity

- SFA- Georgescu-Roegen's bioeconomic model: explaining a production function

Response:

Yes, my dear reviewer, in this section we explain the bioeconomy model with DEA and SFA methodology added.

[17]- “They are essential for the functioning of the economy but aren't always directly accounted for in traditional economic models.” Exact same sentence twice

[26]- Some other production function approaches are listed

Response:

Dear reviewer, it was corrected.

[18]- Bioeconomy model (Equation 17): this is the most interesting section in the whole paper because it finally explains “DEA (Data Envelopment Analysis) and SFA (Stochastic Frontier Analysis) have potential applications in optimizing resource allocation in the bioeconomy.” And what the use of all the presentation of production function was. But there is no application at all. Just some arguments from the literature.

Response:

Dear reviewer, thanks for this comment. Well, this is a different moments of applications, it was discussing in the Discuss Section. However, in DEA and SFA are included the production function that you comment.

[19] Concluding remarks

- Completely unrelated to the whole paper.

Response:

Dear reviewer, it was corrected.
